# Supplementary material for: CircGRIA1 shows an age-related increase in male macaque brain and regulates synaptic plasticity and synaptogenesis
Source: Nat Commun. 2020 Jul 17;11:3594. doi: 10.1038/s41467-020-17435-7 (PMC7367861; doi:10.1038/s41467-020-17435-7)
Supplement: Supplementary file 1 — Supplementary information [file 41467_2020_17435_MOESM1_ESM.pdf]

Supplementary Information

Supplementary Tables

Supplementary Table 1. Sequences of siRNAs and BASEScope probes circGRIA1

circRNA (rhesus macaque)	Sequences of siRNAs and BASEScope assay probes	
Gria1 (rhesus macaque)	junction site (red)	5'-caugauggcauccgaaagggcuucauggacauugac-3'
	siRNA-control 1	3'- gaaagccgaaguaccugua -5'
	siRNA 1	3'- cuuucccgaaguaccugua -5'
	siRNA-control 2	3'- uaccguaggcuuucggcuu -5'
	siRNA 2	3'- uaccguaggcuuucccgaa -5'
	BaseScope™ Target Probe	5'-tgattgaaatgaaacatgatggcatccgaaagggcttcatgg-3' (BA-Mmu-GRIA1-circRNA-Junc.; 1zz targeting 186-227 of the provided sequence >6:150023918|150035468_+)

Supplementary Table 2. Oligos used for circGRIA1 CHIRP in the study

Biotin-circGRIA1-1	guccaugaagcccuuucggaugccaucauguuuc	5'-biotin-labeled oligonucleotide for circGRIA1 pull-down
Biotin-circGRIA1-2	gucaauguccaugaagcccuuucggaugccauc	5'-biotin-labeled oligonucleotide for circGRIA1 pull-down
Biotin-circGRIA1-3	guuuaagucaauguccaugaagcccuuucggau	5'-biotin-labeled oligonucleotide for circGRIA1 pull-down
Biotin-circGRIA1-control-1	gucaauguccaugaagcccagauuugcaaggaug	As control of circGRIA1 pull-down
Biotin-circGRIA1-con-2	caguaaccgaucuuucggaugccaucauguuuc	As control of circGRIA1 pull-down
Biotin-circGRIA1-control-3	cuucauuccaguaaccgaucuuucggaugccauc	As control of circGRIA1 pull-down

Supplementary Figures

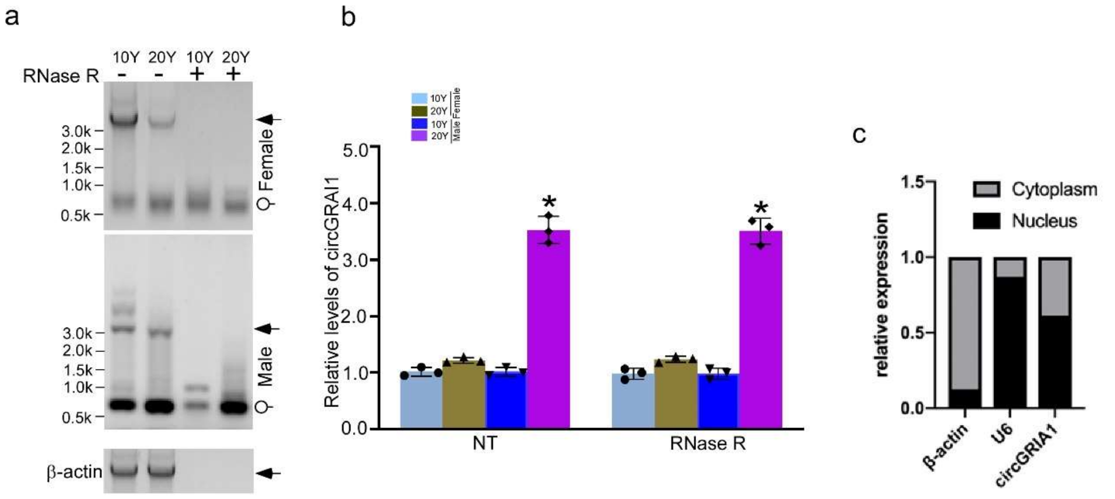

Supplementary Fig 1. Age-related correlation between circGRIA1 and *Gria1* mRNA expression

(**a**) Representative northern blots of Gria1 circular transcripts. Total RNAs were extracted from postmortem frozen hippocampal tissues of 10- and 20-year-old (10Y and 20Y) male macaques, and digested with or without RNase R. Northern blots were performed on the basis of hybridization with the specific Digoxin-labeled *Gria1* single exon 5-targeting probes. K denotes 1,000 nt. The blots are representative of three independent experiment of each sample from 2-3 animals per age group. Notes: Arrow indicates linear transcript, and circle indicates circular transcript. β-actin was loading control.

(b) Relative intensities of northern blot signals illustrated in panel **a**) were quantified by use of image J software. The linear and circular *Gria1* were quantified and calculated in comparison with actin (*, $p < 0.05$, unpaired t-test; mean ± S.D.; n = 3 independent experiment of each sample from 2-3 animals per age group).

(c) CircGRIA1 mainly distributes in the nucleus. Cytoplasmic and nuclear RNAs were prepared from postmortem fresh hippocampal tissues of 20Y male macaque. Extracted RNAs were subjected to RT-qPCR analysis to verify the subcellular localizations of circGRIA1. β-actin was used as the cytoplasm control, and U6 was used as the nucleus control.

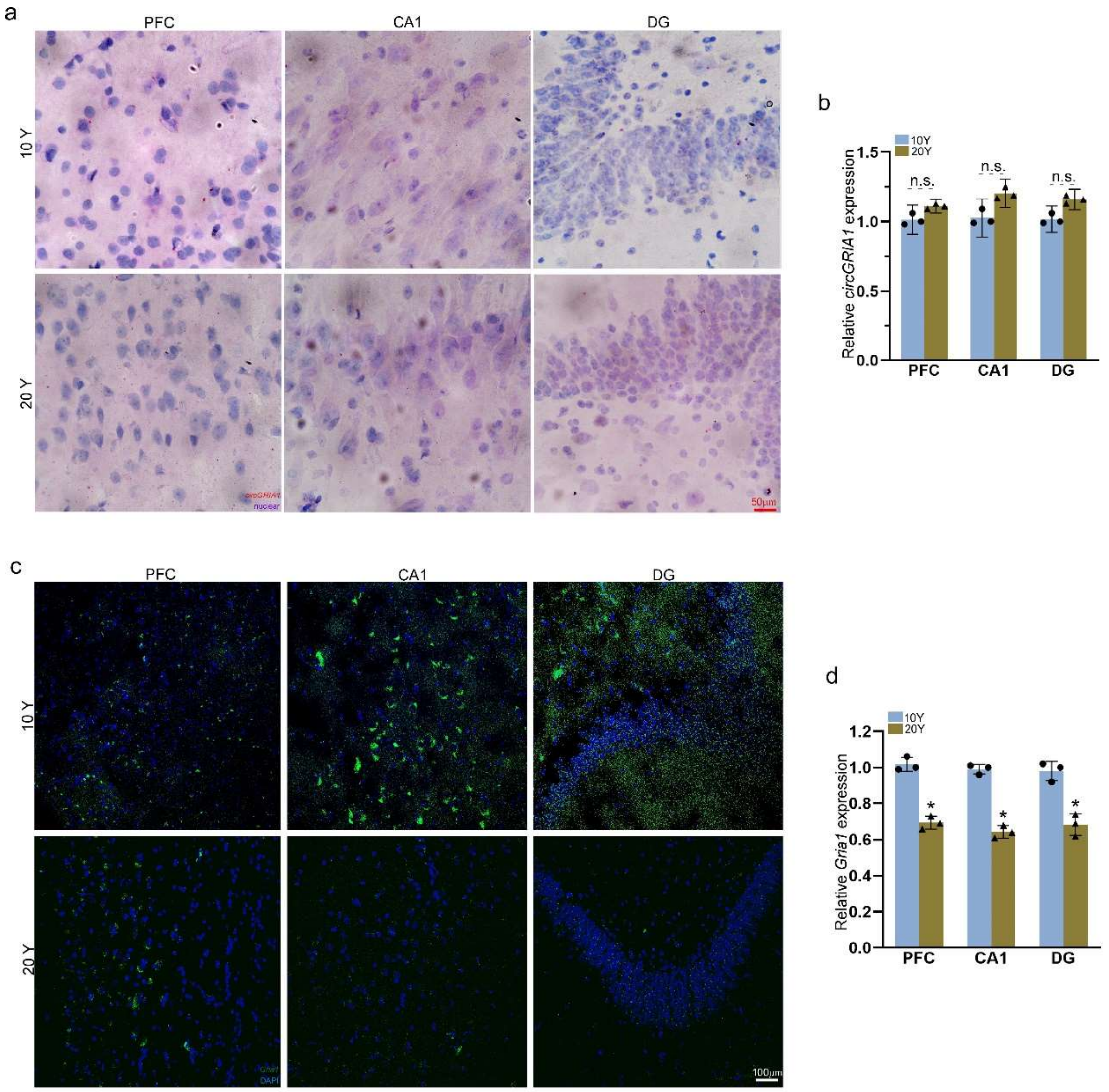

Supplementary Fig 2. No correlation between circGRIA1 and *Gria1* mRNA expression in female macaque brain

(a) BASEscope *in situ* hybridization (ISH) of circGRIA1 in PFC and hippocampus of 10Y and 20Y female macaques. Ten-micron cryostat sections were prepared and performed BASEScope ISH (Red dots with nuclei counterstaining by hematoxylin). Scale bar, 50 μm. The images are representative of three independent experiment of each sample from 2-3 animals per age group.

(b) Relative intensities of BASEscope ISH signals of circGRIA1 illustrated in panel **a**) were quantified by use of Image J. Data are present as mean ± S.D. (n = 35-45 cells per group; *, $p < 0.05$, unpaired t-test).

(c) RNAscope ISH of *Gria1* in PFC and hippocampus of 10Y and 20Y female macaques.

Ten-micron cryostat sections were performed RNAscope ISH (Green dots with nuclei counterstaining by DAPI). Scale bar, 100 μm. White squares represent high magnificent images. The images are representative of three independent experiment of each sample from 2-3 animals per age group

(d) Relative intensities of RNAscope ISH signals of *Grai1* illustrated in panel **c**) were quantified by use of Image J. Data are present as mean ± S.D. (n = 40-50 cells per group; *, $p < 0.05$, unpaired t-test).

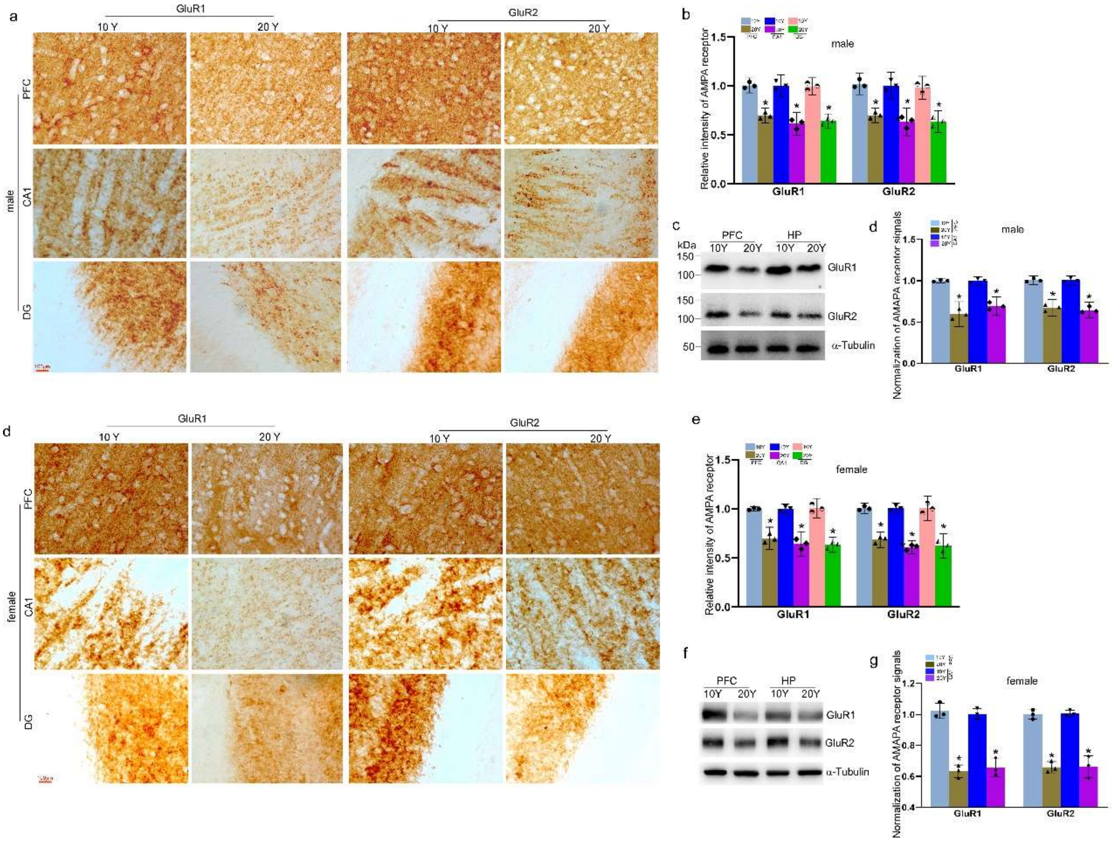

Supplementary Fig 3. GluR1 and GluR2 show an age-related decrease in macaque brain

(a) Representative immunohistochemistry (IHC) images showing changes in levels of GluR1 and GluR2 in PFC and hippocampus of 10Y and 20Y male macaques. Ten-micron cryostat sections were immunostained with GluR1 and GluR2 antibodies using HRP immunocytochemistry (brown). Scale bar, 100 μm.

(b) Relative intensities of GluR1 and GluR2 IHC signals illustrated in panel **a**) were quantified by use of image J software. Data are present as mean ± S.D. Each bar

represents the average of three independent experiments; error bars denote S.D. (*, $p < 0.05$, unpaired *t*-test; $n = 3$ independent experiment of each sample from 2-3 animals per age group).

(c) Protein extracts from postmortem frozen PFC and hippocampal tissues of 10Y and 20Y male macaques were immunoblotted with GluR1 and GluR antibodies. α-Tubulin was loading control. The blots represent three independent experiment of each sample from 2-3 animals per age group.

(d) Relative intensities of immunobloted signals of GluR1 and GluR2 illustrated in panel **c**) were quantified by use of Image J. Data are present as mean ± S.D.; *, $p < 0.05$, unpaired *t*-test.

(e) Representative IHC images showing changes in levels of GluR1 and GluR2 in PFC and hippocampus of 10Y and 20Y female macaques. Ten-micron cryostat sections were immunostained with GluR1and GluR2 antibodies using HRP immunocytochemistry (brown). Scale bar, 100 μm.

(f) Relative intensities of GluR1 and GluR2 IHC signals illustrated in panel **e**) were quantified by use of image J software. Data are present as mean ± S.D. Each bar represents the average of three independent experiment; error bars denote S.D. (*, $p < 0.05$, unpaired *t*-test; $n = 3$ independent experiment of each sample from 2-3 animals per age group).

(g) Protein extracts from postmortem frozen PFC and hippocampal tissues of 10Y and 20Y female macaques were immunoblotted with GluR1 and GluR2 antibodies. α-Tubulin was loading control. The blots represent three independent experiment of each sample from 2-3 animals per age group.

(h) Relative intensities of immunobloted signals of GluR1 and GluR2 illustrated in panel **c**) were quantified by use of Image J. Data are present as mean ± S.D.; *, $p < 0.05$, unpaired *t*-test.

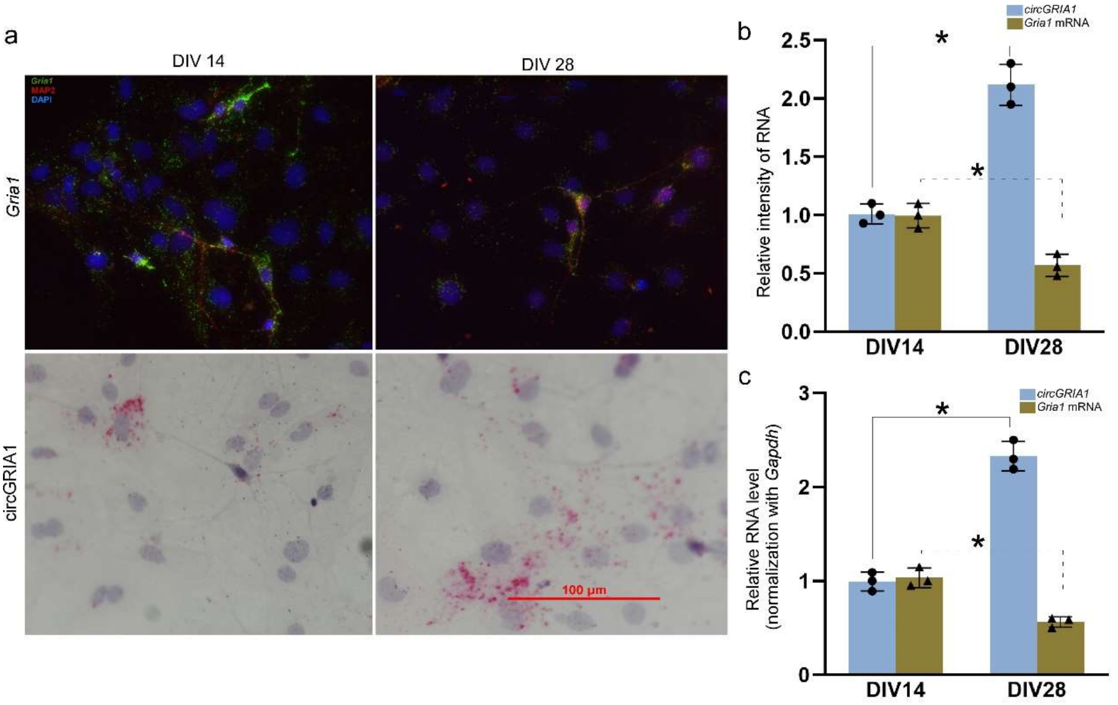

Supplementary Fig 4. Hippocampal neurons of the fetal macaque reproduce the neurobiology of aging

(a) Hippocampal cultures of male fetal macaque were prepared as indicated time. Neurons were fixed and were performed BASEscope ISH (Red dots with nuclei counterstaining by hematoxylin) and RNAscope ISH (Green dots with nuclei counterstaining by DAPI) at DIV 14 and DIV 28. Scale bar, 100 μm. The images are representative of three independent experiment of each hippocampal culture from 2-3 fetal macaques.

(b) Relative intensities of circGRIA1 and *Gria1* ISH signals per neuron illustrated in **a**) were quantified by use of Image J. Data are present as mean ± S.D. (n = 25-30 per fetal culture; *, $p < 0.01$, unpaired t-test).

(c) Total RNAs were extracted from DIV14 and DIV28 hippocampal cultures of male fetal macaques, and performed RT-qPCR for validation circGRIA1 and its host gene *Gria1* mRNA expression. Data are presented as mean ± S.D.; n = 3 independent experiment of each hippocampal culture from 2-3 fetal macaques; *, $p < 0.05$, unpaired t-test.

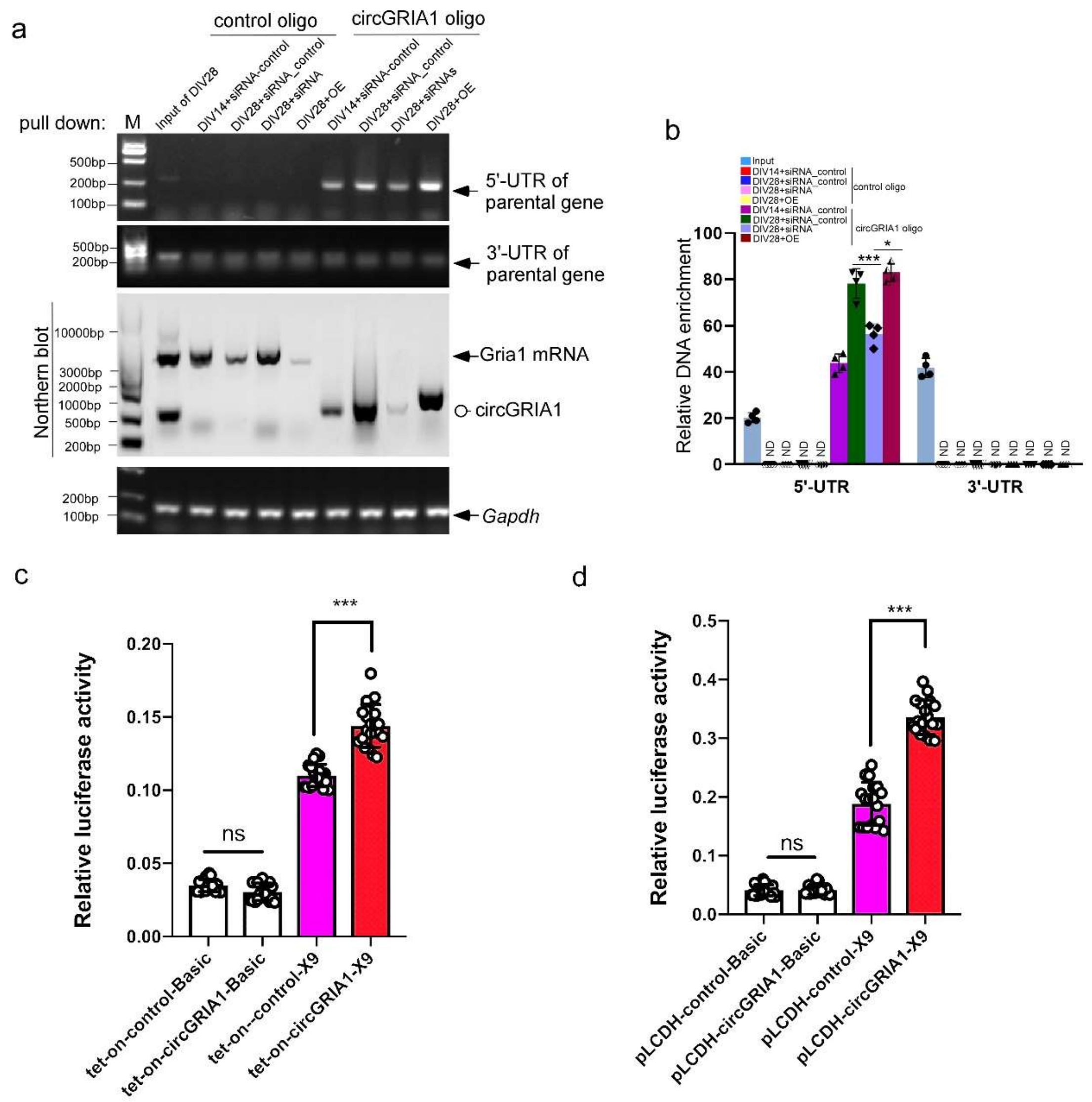

Supplementary Fig 5. The association of circGRIA1 with the promoter region of its parental gene

(a) Hippocampal cultures of male fetal macaque were infected at DIV5 with viral circGRIA1, or siRNAs against circGRIA1 or siRNA-control. At DIV14 and DIV28, neurons were collected, and performed CHIRP with biotin-labeled oligos complementary to the junction sequences of circGRIA1 followed by PCR and Northern blot. Upper panel shows PCR products of the parental gene 5'-UTR and 3'-UTR regions against circGRIA1 oligos in ChIRP. Middle panel shows northern blot of *Gria1* mRNA and circGRIA1 in the product of ChIRP with Digoxin-labeled probes. Semi-quantitative RT-PCR of *Gapdh* was a loading control.

(b) Quantification of three independent experiment of each hippocampal culture from

2-3 fetal male macaques in **a)**. Data represents mean ± S.D.; *, $p < 0.05$; ***, $p < 0.001$, unpaired *t*-test.

(c) The relative luciferase activities of luciferase reporters containing 5′UTR sequence (~330bp) of macaque *Gria1* in pGL4.11 vector, and SH-SY5Y cells transfected with circGRIA1 in Tet-on circRNA vector. Results are presented as mean ± S.D. (***$p < 0.01$).

(d) The relative luciferase activities of luciferase reporters containing 5′UTR sequence (~330bp) of macaque *Gria1*in pGL4.11 vector, and SH-SY5Y cells transfected with circGRIA1 in pLCDH-ciR vector. Results are presented as mean ± S.D. (***$p < 0.01$, n=10-15 repetitions of independent experiment).

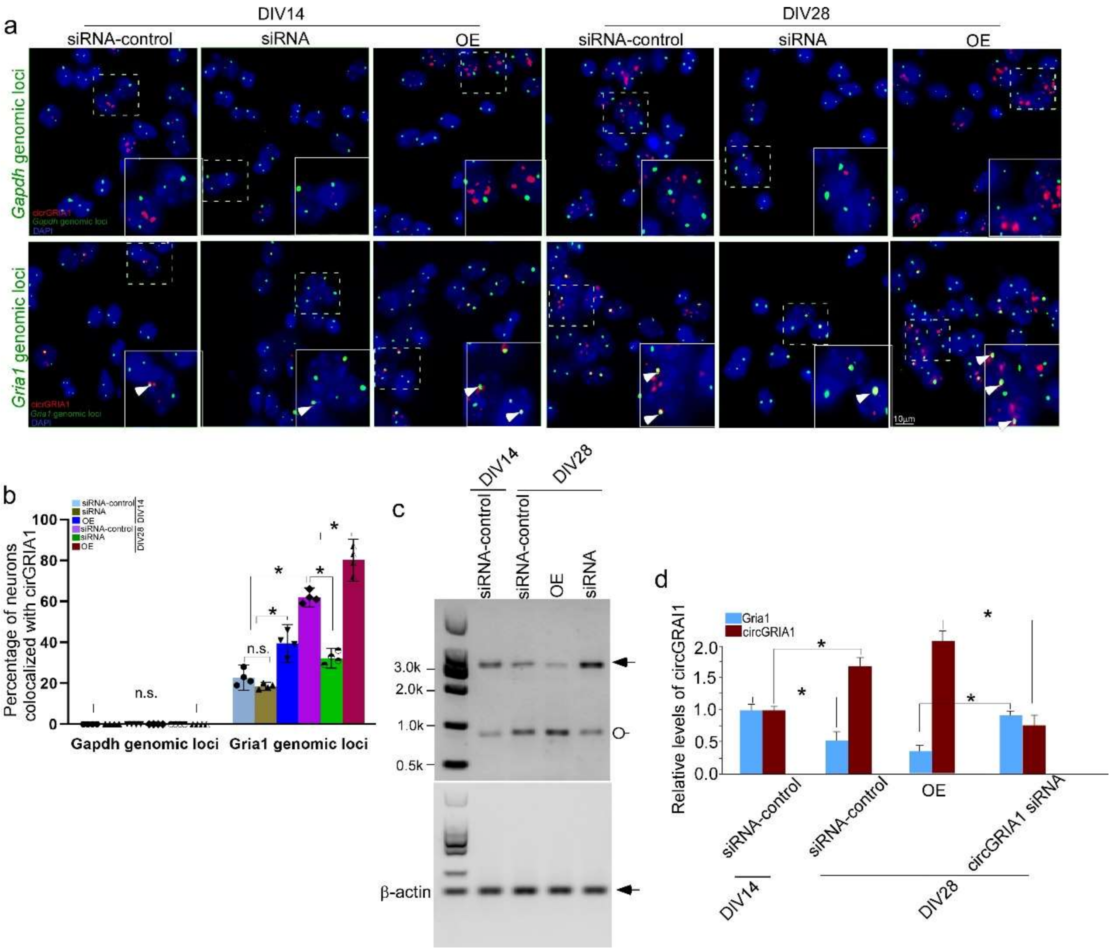

Supplementary Fig 6. The association of increased nuclear circGRIA1 with its parental gene loci

(a) The association of circGRIA1 with parental gene loci over the aging states.

Hippocampal cultures of male fetal macaque were infected at DIV5 with viral particles of circGRIA1, or the junction site-targeting siRNAs against circGRIA1 or the mismatched junction site-targeting siRNA-control. At DIV14 and DIV28, neurons were fixed and were performed dual FISH. Left panel, representative double FISH images for circGRIA1 and parental gene loci (n = 66-79 for circGRIA1) showing colocalization of circGRIA1 (detected with a junction probe, Red dots) and the corresponding parental gene loci (Green dots with nuclei counterstaining by DAPI). Right panel, representative dual FISH images (n = 39-45 cells for each image panel) showing no circGRIA1 colocalization with the genomic loci of *Gapdh.* The circGRIA1 FISH signal was detected with a junction probe. Scale bars, 10 μm.

(b) Relative intensities of dual FISH staining signal illustrated in panel **a**) were quantified by use of image J software. Statistical analysis for the culture states is shown in bar graph. The parental gene loci are colocalized with the circGRIA1 in neurons at DIV28 more than that of at DIV14, and the percentages are ~30% for DIV14 and ~65 % for DIV28 (n = 66 cells for DIV14 and 79 cells for DIV28). Data are present as mean ± S.D.; n.s., no significance; *, $p < 0.01$, unpaired t-test. Three independent experiment were performed in each hippocampal culture from 2-3 fetal male macaques.

(c) Hippocampal cultures of male fetal macaque were infected at DIV5 with viral circGRIA1, or siRNAs against circGRIA1 or siRNA-control. At DIV14 and DIV28, neurons were collected, and performed with biotin-labeled oligos complementary to the junction sequences of circGRIA1 followed Northern blot. Notes: Arrow indicates linear transcript, and circle indicates circular transcript. β-actin was loading control.

(d) Quantification of three independent experiment of each hippocampal culture from 2-3 fetal male macaques in **c**). Data represents mean ± S.D.; *, $p < 0.05$; unpaired t-test.

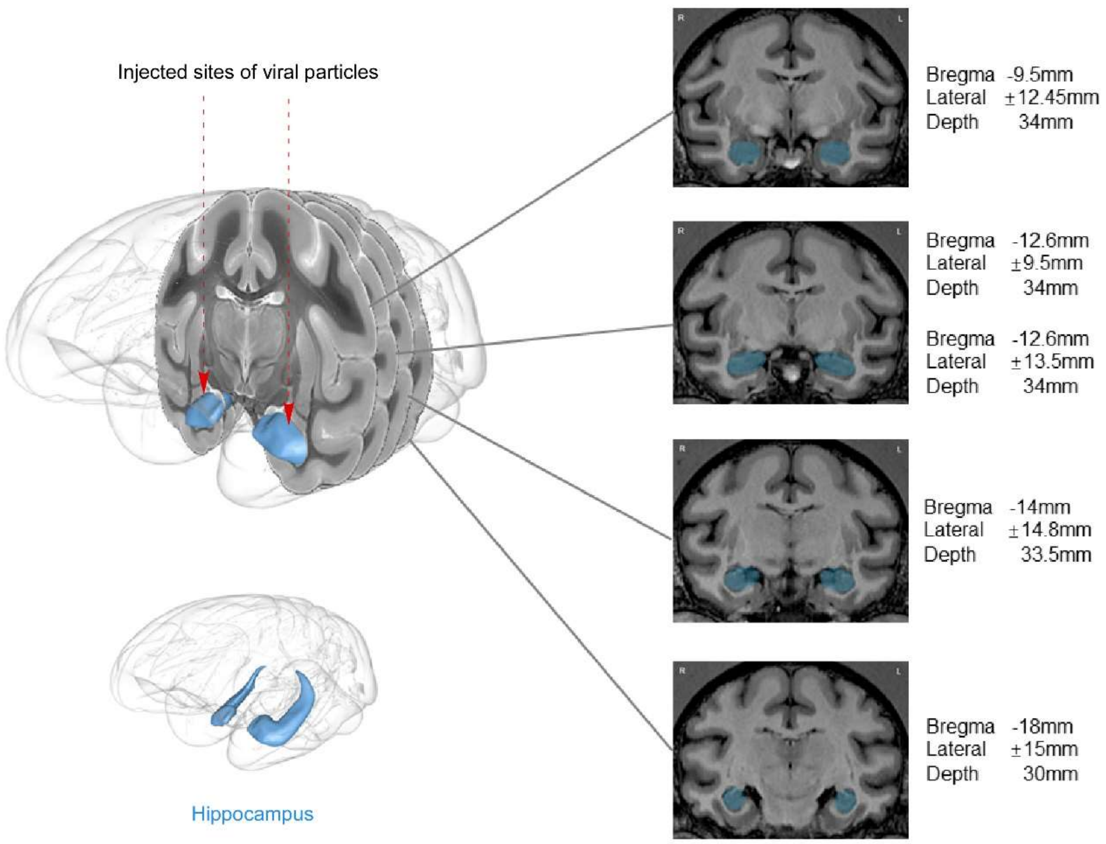

Supplementary Fig. 7. Schematic map of the microinjection sites of viral particles
3.0 T MRI (uMR770, United Imaging, China) scanning was conducted to determine the location of microinjection of viral particles into hippocampus of macaque with a circular 12-chanel coil. Left parts was the template from The Scalable Macaque Brain Atlas with modification (Author copy: arXiv:1312.6310)[1,2], and right parts were the coronal brain MR images with defined microinjection sites in macaques used in this study.

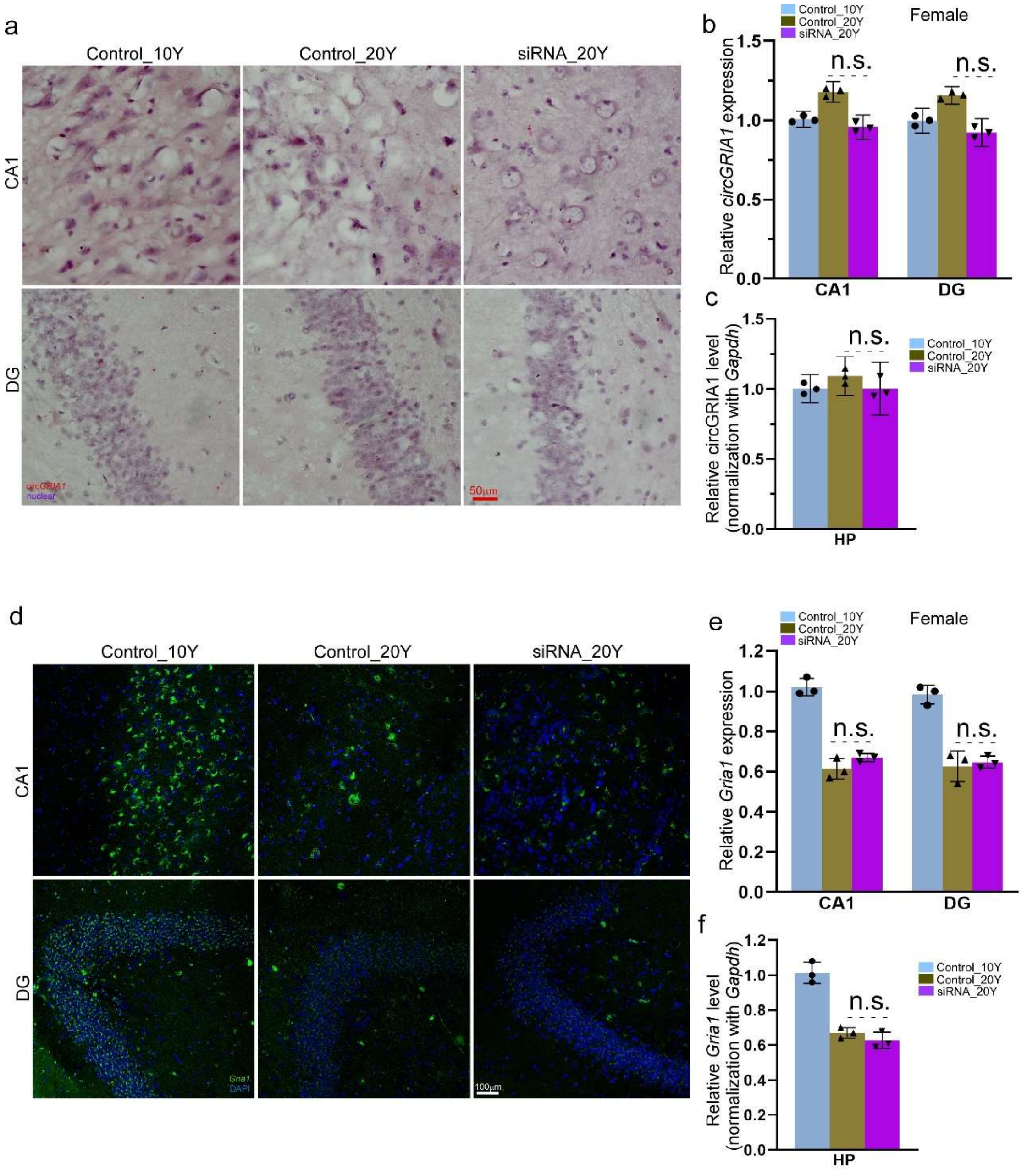

Supplementary Fig. 8. Knockdown of circGRIA1 shows little effect on *Gria1* mRNA expression in female neurons

(a) Representative images of BASEscope ISH showing circGRIA1 expression in hippocampus of 10Y and 20Y female macaques. Viral particles containing siRNAs against circGRIA1 or siRNA-control were microinjected into hippocampus of macaque as indicated. Six weeks after operation, animals were anesthetized, brains were then removed rapidly and fixed with 4% paraformaldehyde. Hippocampal ten-micron cryostat sections were prepared and performed BASEscope ISH (Red dots with nuclei counterstaining by hematoxylin). Scale bar, 50 μm. The images are representative of

three independent experiment of each sample from 2-3 animals per group.

(b) Relative intensities of BASEscope ISH signals of circGRIA1 illustrated in panel **a**) were quantified by use of Image J. Data are present as mean ± S.D. (*n* = 25-35 cells per group; n.s., no significance, unpaired *t*-test).

(c) Total RNAs were extracted from frozen hippocampal tissues of 10Y and 20Y female macaques with microinjection of viral particles containing siRNAs against circGRIA1 or siRNA-control into hippocampus of macaque as indicated. RT-qPCR was performed for validation of circGRIA1 expression. Data are presented as mean ± S.D.; n = three independent experiments from three animals per age group; n.s, no significance; unpaired *t*-test.

(d) Representative images of RNAscope ISH showing *Gria1* expression in hippocampus of 10Y and 20Y female macaques. Hippocampal ten-micron cryostat sections were performed RNAScope ISH (Green dots with nuclei counterstaining by DAPI). Scale bar, 100 μm. The images are representative of three independent experiment of each sample from 2-3 animals per group.

(e) Relative intensities of RNAscope ISH signals of *Grai1* illustrated in panel **d**) were quantified by use of Image J. Data are present as mean ± S.D. (*n* = 50-55 cells per group; n.s., no significance, unpaired *t*-test).

(f) Total RNAs were extracted from frozen hippocampal tissues of 10Y and 20Y female macaques with microinjection of viral particles containing siRNAs against circGRIA1 or siRNA-control into hippocampus of macaque as indicated. RT-qPCR was performed for validation of *Gria1* expression. Data are presented as mean ± S.D.; n = three independent experiment of each sample from 2-3 animals per age group; n.s, no significance; unpaired *t*-test.

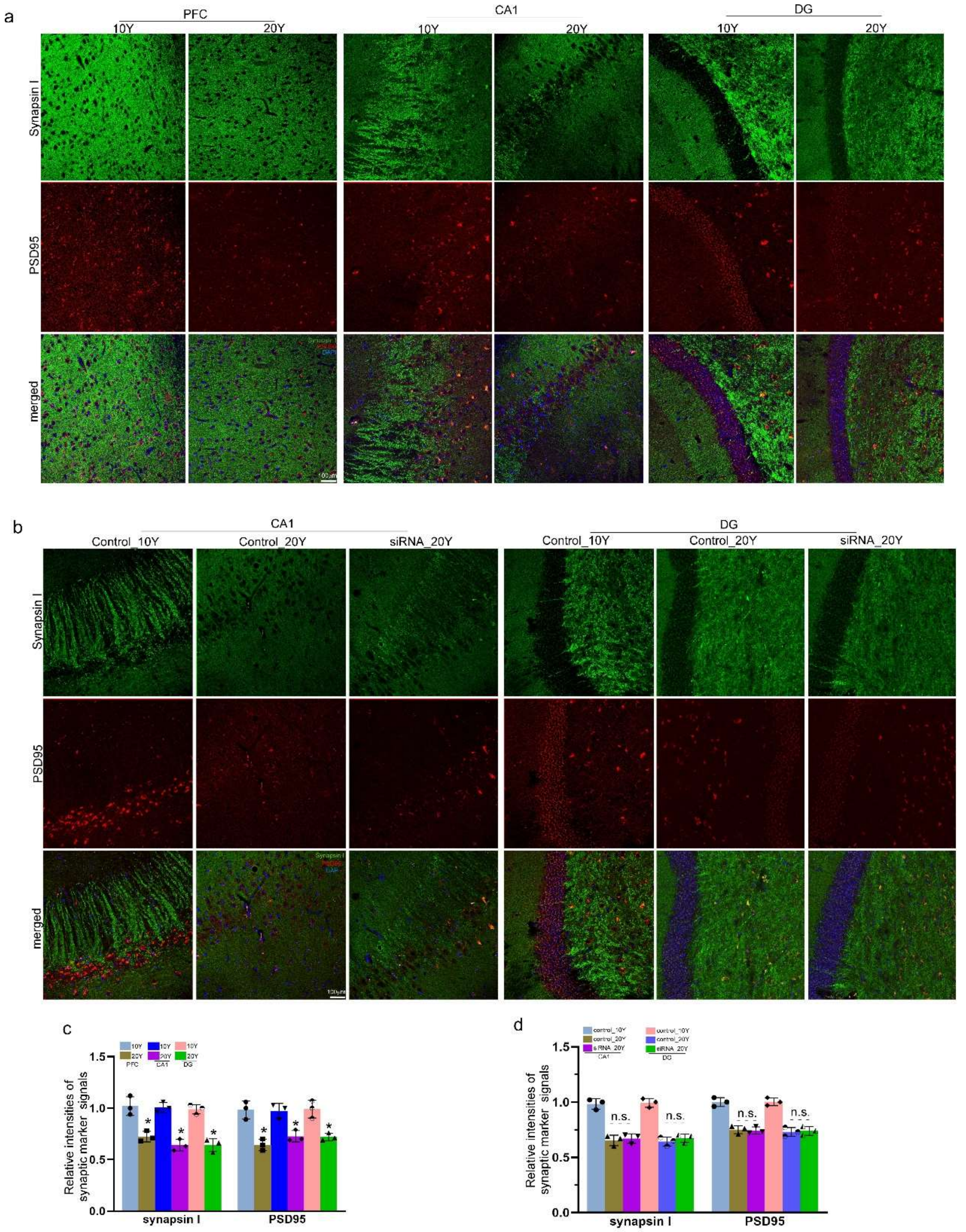

Supplementary Fig. 9. Knockdown of circGRIA1 little affects synaptogenesis in hippocampal neurons of female macaques

(a) Representative images of immunofluorescence (IF) showing levels of synapsin-I and PSD95 in PFC and hippocampal (CA1 and DG) of 10Y and 20Y female macaques. Ten-micron cryostat sections were immunostained with synapsin-I (Green) and PSD95 (Red) antibodies. Scale bar, 100 μm.

(b) Representative images of IF showing levels of synapsin-I and PSD95 in hippocampus of 10Y and 20Y female macaques after *in vivo* knockdown of circGRIA1. Hippocampal ten-micron cryostat sections were immunostained with synapsin-I (Green) and PSD95 (Red) antibodies. Scale bar, 100 μm.

(c) Relative intensities of synapsin-I and PSD95 IF signals illustrated in panel **a**) were quantified by use of Image J. Data are present as mean ± S.D. (n = 2-3 animals per age group). Each bar represents the average of three independent experiment; error bars denote S.D. (*, $p < 0.05$, unpaired t-test).

(d) Relative intensities of synapsin-I and PSD95 immunostaining illustrated in panel **c**) were quantified by use of Image J. Data are present as mean ± S.D. (n = 2-3 animals per age group). Each bar represents the average of three independent experiment; error bars denote S.D. (n.s., no significance, unpaired t-test).

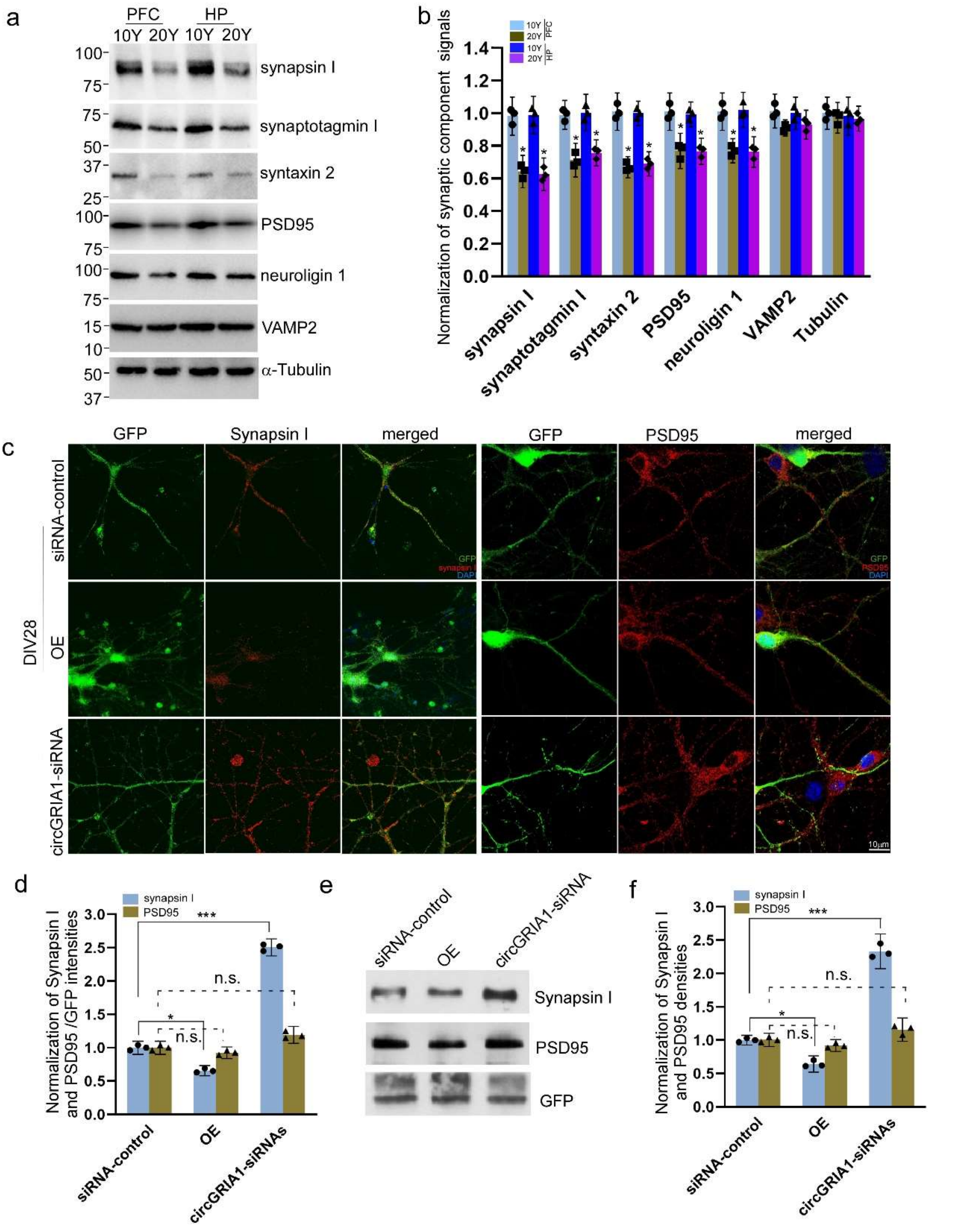

Supplementary Fig. 10. CircGRIA1 regulates synaptogenesis

(a) Protein extracts from postmortem frozen PFC and hippocampal tissues of 10Y and 20Y male macaques were immunoblotted with synaptic component antibodies as indicated. α-Tubulin was loading control. The blots represent three independent experiment of each sample from 2-3 animals per age group.

(b) Relative intensities of immunobloted signals of synaptic components illustrated in

panel **a**) were quantified by use of Image J. Data are present as mean ± S.D.; *, $p < 0.05$, unpaired *t*-test.

(c) Hippocampal cultures of male fetal macaque were infected at DIV5 with viral particles containing GFP and circGRIA1, or the junction site-targeting siRNAs against circGRIA1 or the mismatched junction site-targeting siRNA-control. At DIV28, neurons were fixed and stained with either synapsin-I (Red) or PSD95 (Red) antibodies. Scale bar, 10 μm.

(d) Normalized intensities of synapsin-I or PSD95 immunostaining as compared to GFP illustrated in panel **c**) were quantified by use of image J software. Data are present as mean ± S.D. (*n* = 26-38 cells per group). Each bar represents the average of three independent experiment of each hippocampal culture from 2-3 fetal macaques; n.s., no significance; *, $p < 0.01$; ***, $p < 0.005$, unpaired *t*-test.

(e) Protein extracts from DIV28 hippocampal cultures of male fetal macaque infected at DIV5 with viral circGRIA1, or siRNAs against circGRIA1 or siRNA-control, were immunoblotted with synapsin I and PSD95 antibodies. GFP was loading control. The blots represent three independent experiment of each culture from 2-3 fetal macaques.

(f) Relative intensities of immunobloted signals of synapsin-I and PSD95 illustrated in panel **e**) were quantified by use of Image J. Data are present as mean ± S.D. Each bar represents the average of three independent experiment of each culture from 2-3 fetal macaques; error bars denote S.D.; n.s., no significance; *, $p < 0.05$,

Supplementary References:

1 Bakker, R., Tiesinga, P. & Kotter, R. The Scalable Brain Atlas: Instant Web-Based Access to Public Brain Atlases and Related Content. *Neuroinformatics* **13**, 353-366, doi:10.1007/s12021-014-9258-x (2015).

2 Calabrese, E. *et al.* A diffusion tensor MRI atlas of the postmortem rhesus macaque brain. *Neuroimage* **117**, 408-416, doi:10.1016/j.neuroimage.2015.05.072 (2015).
