# Supplementary material for: CircGRIA1 shows an age-related increase in male macaque brain and regulates synaptic plasticity and synaptogenesis
Source: Nat Commun. 2020 Jul 17;11:3594. doi: 10.1038/s41467-020-17435-7 (PMC7367861; doi:10.1038/s41467-020-17435-7)
Supplement: Supplementary file 3 — Description of Additional Supplementary Files [file 41467_2020_17435_MOESM3_ESM.pdf]

Description of Additional Supplementary Files

File Name: Supplementary Data 1
Description: Bioinformatic data of circGRIA1.

File Name: Supplementary Data 2
Description: Comparable analysis of circGRIA1 in human, mouse and macaque.

File Name: Supplementary Data 3
Description: The primers for qPCR and northern blot probes.
