# Supplementary material for: CircGRIA1 shows an age-related increase in male macaque brain and regulates synaptic plasticity and synaptogenesis
Source: Nat Commun. 2020 Jul 17;11:3594. doi: 10.1038/s41467-020-17435-7 (PMC7367861; doi:10.1038/s41467-020-17435-7)
Supplement: Supplementary file 7 — Reporting Summary [file 41467_2020_17435_MOESM7_ESM.pdf]

nature research

Corresponding author(s): Jiali Li

Last updated by author(s): 04/28/2020

Reporting Summary

Nature Research wishes to improve the reproducibility of the work that we publish. This form provides structure for consistency and transparency in reporting. For further information on Nature Research policies, seeAuthors & Referees and theEditorial Policy Checklist .

Statistics

For all statistical analyses, confirm that the following items are present in the figure legend, table legend, main text, or Methods section.

n/a	Confirmed	
☐	☒	The exact sample size (*n*) for each experimental group/condition, given as a discrete number and unit of measurement
☐	☒	A statement on whether measurements were taken from distinct samples or whether the same sample was measured repeatedly
☐	☒	The statistical test(s) used AND whether they are one- or two-sided<br>*Only common tests should be described solely by name; describe more complex techniques in the Methods section.*
☒	☐	A description of all covariates tested
☐	☒	A description of any assumptions or corrections, such as tests of normality and adjustment for multiple comparisons
☐	☒	A full description of the statistical parameters including central tendency (e.g. means) or other basic estimates (e.g. regression coefficient) AND variation (e.g. standard deviation) or associated estimates of uncertainty (e.g. confidence intervals)
☐	☒	For null hypothesis testing, the test statistic (e.g. *F*, *t*, *r*) with confidence intervals, effect sizes, degrees of freedom and *P* value noted<br>*Give P values as exact values whenever suitable.*
☒	☐	For Bayesian analysis, information on the choice of priors and Markov chain Monte Carlo settings
☒	☐	For hierarchical and complex designs, identification of the appropriate level for tests and full reporting of outcomes
☒	☐	Estimates of effect sizes (e.g. Cohen's *d*, Pearson's *r*), indicating how they were calculated

Our web collection on statistics for biologists contains articles on many of the points above.

Software and code

Policy information about availability of computer code

Data collection	No software was used other than those listed in the Methods section.
Data analysis	Find_circ, CIRI2, Image J v1.8.0, Prism 7.0(GraphPad), microsoft Excel (2016). The circRNA isoforms from the genomic locus of Gria1 by find_circ and CIRI2 methods. mEPSCs were analyzed offline using Stimfit software by employing a template-matching algorithm. The intensity of intracellular Calcium fluorescence of single neurons was individually measured by the NIS Elements AR 3.2 Software (Nikon). MRI data was analyzed using Brainsight system.

For manuscripts utilizing custom algorithms or software that are central to the research but not yet described in published literature, software must be made available to editors/reviewers. We strongly encourage code deposition in a community repository (e.g. GitHub). See the Nature Research guidelines for submitting code & software for further information.

Data

Policy information about availability of data

All manuscripts must include a data availability statement. This statement should provide the following information, where applicable:

- Accession codes, unique identifiers, or web links for publicly available datasets
- A list of figures that have associated raw data
- A description of any restrictions on data availability

The data has been described in our previous study(Xu et al., 2018, Cell Discovery), and deposited in GEO and are accessible through accession number GSE94027; Figure 1, and Supplementary Table 1.

Field-specific reporting

Please select the one below that is the best fit for your research. If you are not sure, read the appropriate sections before making your selection.

- [x] Life sciences
- [ ] Behavioural & social sciences
- [ ] Ecological, evolutionary & environmental sciences

For a reference copy of the document with all sections, see nature.com/documents/nr-reporting-summary-flat.pdf

Life sciences study design

All studies must disclose on these points even when the disclosure is negative.

Sample size	No sample size calculation was applied in advance.
Data exclusions	No data was excluded in this study.
Replication	All code and data used in this study are made openly available for reproducibility
Randomization	We allocated samples into experimental groups by their age /sex or indicated time. The monkey postmortem brain samples within each group were randomly chosen.
Blinding	The investigators were not blinded during data acquisition.

Reporting for specific materials, systems and methods

We require information from authors about some types of materials, experimental systems and methods used in many studies. Here, indicate whether each material, system or method listed is relevant to your study. If you are not sure if a list item applies to your research, read the appropriate section before selecting a response.

Materials & experimental systems

n/a	Involved in the study	
[ ]	[x]	Antibodies
[ ]	[x]	Eukaryotic cell lines
[x]	[ ]	Palaeontology
[ ]	[x]	Animals and other organisms
[x]	[ ]	Human research participants
[x]	[ ]	Clinical data

Methods

n/a	Involved in the study	
[x]	[ ]	ChIP-seq
[x]	[ ]	Flow cytometry
[ ]	[x]	MRI-based neuroimaging

Antibodies

Antibodies used	Antibodies against GluR1 (ab31232), GluR2 (ab133477), GFP(ab1213, ab183734), PSD95 (ab2723, ab13552), synapsin I (ab64581), syntaxin 2 (ab233275), synaptotagmin (ab13259), VAMP2 (ab181869), neuroligin 1 (ab153821), NR2A (ab14596), NR2B (ab65783), alpha-tubulin (ab7291) and MAP2 (ab5392) were purchased from Abcam. Secondary antibodies used for immunocytochemistry were: Alexa 488-labeled chicken anti-mouse or anti-rabbit; Alexa 594-labeleddonkey anti-mouse or anti-rabbit (Invitrogen, Eugene, OR).
Validation	All the antibodies were validated for use in mouse/human /monkey tissues. Data are available on the manufacturer's websites.

Eukaryotic cell lines

Policy information about cell lines

Cell line source(s)	Human neuroblastoma cells (SH-SY5Y) were used in this study.
Authentication	SH-SY5Y cells (KCB2006107YJ) were obtained from Kunming Cell Bank, Chinese Academe of Sciences.
Mycoplasma contamination	SH-SY5Y cells were tested negative for mycoplasma.
Commonly misidentified lines (See ICLAC register)	No commonly misidentified cell lines were used.

Animals and other organisms

Policy information about studies involving animals; ARRIVE guidelines recommended for reporting animal research

Laboratory animals	Wild type male rhesus monkey specimens at each of stages representing adult (10-year old) and old (20-year old) were profiled as our previous report (Xu et al., 2018, Cell Discovery). The detailed information of animal age and sex was described in each experiment. Wild type male and female monkey fetus (15 weeks) were used for primary hippocampal neurons. Adult and old macaques in both genders were utilized in this study.
Wild animals	Not applicable.
Field-collected samples	No field-collected samples were used in this study.
Ethics oversight	All animal experiments were conducted with the license from Kunming Institute of Zoology, and were approved by the Animal Care and Use Committee of Kunming Institute of Zoology, Chinese Academy of Sciences.

Note that full information on the approval of the study protocol must also be provided in the manuscript.

Magnetic resonance imaging

Experimental design

Design type	Structural MRI under anesthesia.
Design specifications	MRI data are used for stereotaxic and neuronavigation.
Behavioral performance measures	No behavioral performance is reported.

Acquisition

Imaging type(s)	T1-weighted and T2-weighted MRI
Field strength	3.0 T
Sequence & imaging parameters	This information is detailed in the manuscript.
Area of acquisition	Whole brain scan.

Diffusion MRI ☐ Used ☒ Not used

Preprocessing

Preprocessing software	Brainsight.
Normalization	Data was not normalized.
Normalization template	Data was not aligned to any standard template.
Noise and artifact removal	The physiological noise correction was implemented through an anatomical component based noise correction approach.
Volume censoring	None.

Statistical modeling & inference

Model type and settings	None.
Effect(s) tested	None.

Specify type of analysis: ☒ Whole brain ☐ ROI-based ☐ Both

Statistic type for inference (See Eklund et al. 2016)	None.
Correction	None.

Models & analysis

n/a	Involved in the study
☒	☐ Functional and/or effective connectivity
☒	☐ Graph analysis
☒	☐ Multivariate modeling or predictive analysis
